# Facial esthetics and the assignment of personality traits before and after orthognathic surgery rated on video clips

**DOI:** 10.1371/journal.pone.0191718

**Published:** 2018-02-01

**Authors:** Klaus Sinko, Reinhold Jagsch, Claudio Drog, Wilhelm Mosgoeller, Arno Wutzl, Gabriele Millesi, Clemens Klug

**Affiliations:** 1 Clinic for Cranio-Maxillofacial and Oral Surgery, Medical University Vienna, Vienna, Austria; 2 Clinical Psychology and Health Psychology, Department of Psychology, University of Vienna, Vienna, Austria; 3 University Clinic of Dentistry, Medical University Vienna, Vienna, Austria; 4 Institute of Cancer Research, Medical University Vienna, Vienna, Austria; University Hospital Muenster, GERMANY

## Abstract

Typically, before and after surgical correction faces are assessed on still images by surgeons, orthodontists, the patients, and family members. We hypothesized that judgment of faces in motion and by naïve raters may closer reflect the impact on patients’ real life, and the treatment impact on e.g. career chances. Therefore we assessed faces from dysgnathic patients (Class II, III and Laterognathia) on video clips. Class I faces served as anchor and controls. Each patient’s face was assessed twice before and after treatment in changing sequence, by 155 naïve raters with similar age to the patients. The raters provided independent estimates on aesthetic trait pairs like ugly /beautiful, and personality trait pairs like dominant /flexible. Furthermore the perception of attractiveness, intelligence, health, the persons’ erotic aura, faithfulness, and five additional items were rated. We estimated the significance of the perceived treatment related differences and the respective effect size by general linear models for repeated measures. The obtained results were comparable to our previous rating on still images. There was an overall trend, that faces in video clips are rated along common stereotypes to a lesser extent than photographs. We observed significant class differences and treatment related changes of most aesthetic traits (e.g. beauty, attractiveness), these were comparable to intelligence, erotic aura and to some extend healthy appearance. While some personality traits (e.g. faithfulness) did not differ between the classes and between baseline and after treatment, we found that the intervention significantly and effectively altered the perception of the personality trait self-confidence. The effect size was highest in Class III patients, smallest in Class II patients, and in between for patients with Laterognathia. All dysgnathic patients benefitted from orthognathic surgery. We conclude that motion can mitigate marked stereotypes but does not entirely offset the mostly negative perception of dysgnathic faces.

## Introduction

An attractive face correlates with a number of positively perceived traits [1], which can impact upon personal relationships [2]. Aspects like symmetry, averageness or a baby face influence the observer, regardless of his/her cultural background [3]. A retruded chin is typically perceived as a baby face and conveys social submissiveness [4]. Livingston and Pearce [5] have shown that facial traits can influence careers and financial success. Also political leadership correlates with the so called "power face" which is leptoprosopic [6]. So called skeletal Class I faces are generally considered more attractive than Class II and/or Class III faces [4, 7–9]. Whilst in the first half of the last century a slightly retruded chin was considered a female beauty ideal, a straight profile with a rather dominant chin has been deemed aesthetic since the 1980s. However, it remains a matter of debate whether Class II [8, 10], or Class III [4, 11] is the less attractive.

If we meet an unfamiliar person the first impression is captured subconsciously and based on rather meagre information [12]. Faces presented in videos provide more information to the observers’ unconscious assessment of aesthetic and personality traits than still images. Rhodes [13] maintains that facial averaging and symmetry render faces, more attractive in both settings: videos and photographs. Rubenstein [14] in contrast, claims faces more attractive in video recordings, and less attractive in photographs. O’Toole *et al*. [15] found that faces and bodies in motion were perceived and rated more favorably.

Orthognathic surgery improves both, function and aesthetics [16], and thereby, improves the patients’ psychosocial situation [17]. When dysgnathic patients seek the help of maxillofacial surgeons, the aim of the surgical intervention is to change their facial appearance towards the generally accepted “benchmark” the skeletal Class I face. The key challenge for the surgeon is to optimize perception by persons that are not already a part of the patient’s social network.

Orthognathic surgery treatment strategies are complex and individualized. Before and after any surgical procedure, faces are normally assessed and studied by photographs (still images). However, in real life faces are not still, their expression is under constant influence by muscle function related to speech and reflecting emotions.

Because moving images (video clips) may convey more relevant aesthetics/personality related information than photographs and, thereby, ultimately allow the planning of the aesthetics aspects of orthognathic surgery to be further optimized we tested how typical aesthetic traits and—in addition to this—the perception of personal traits in dysgnathic patients change due to orthognathic surgery.

## Material and methods

### Patients & videos

Our clinic is a central hospital for the east Austrian region, with approximately 100 surgical corrections of dysgnathia per year. This study was ethically approved by the Ethic commission of the Medical University Vienna/Austria (File No: 1758/2015). We invited dysgnathic patients to participate in the study and allow us the production of a standardized videoclip before and after treatment. Each participating patient provided written informed consent and allowed their videoclip to be presented to raters for medical teaching or scientific purposes. For publication we followed the rules of the US guidance regarding methods for de-identification of protected health information in accordance with the health insurance portability and accountability act (HIPAA) privacy rule or obtained written consent for publication. There were no further specific inclusion criteria. The patients received no compensation for their participation.

A clinical expert team (KS, CD, CK) selected the videos for presentation to the raters from the pool of patients that had provided a video before and after the treatment. Care was taken to select video pairs from patients with a marked dysgnathia. Furthermore to standardize the presentations, the videos were taken from the probands (dysgnathic patients and controls) without extensive makeup, in uniform clothing (green operating room shirt and a green surgical cap) in front of a black background. Patients’ videos with smooth movements were given preference over videos with distracting and diverting contents. Four video pairs from controls (Class I) and 4 video pairs from each of 3 patients groups (Class II, Class III and Laterognathia) were selected. From the twelve patients 10 patients were subjected to bi-maxillary surgery, two patients (Class II) received Bilateral Sagittal Split Osteotomy (BSSO). We compared patient video recordings, taken before and after the complete surgical and orthodontic treatment.

The four controls (Class I) were picked from the social environment of the authors. Their age was matched to the patients’ age. To match for possible signs of aging the second video was produced roughly with the same time difference as for the patients’ mean treatment time.

**Fig 1:**
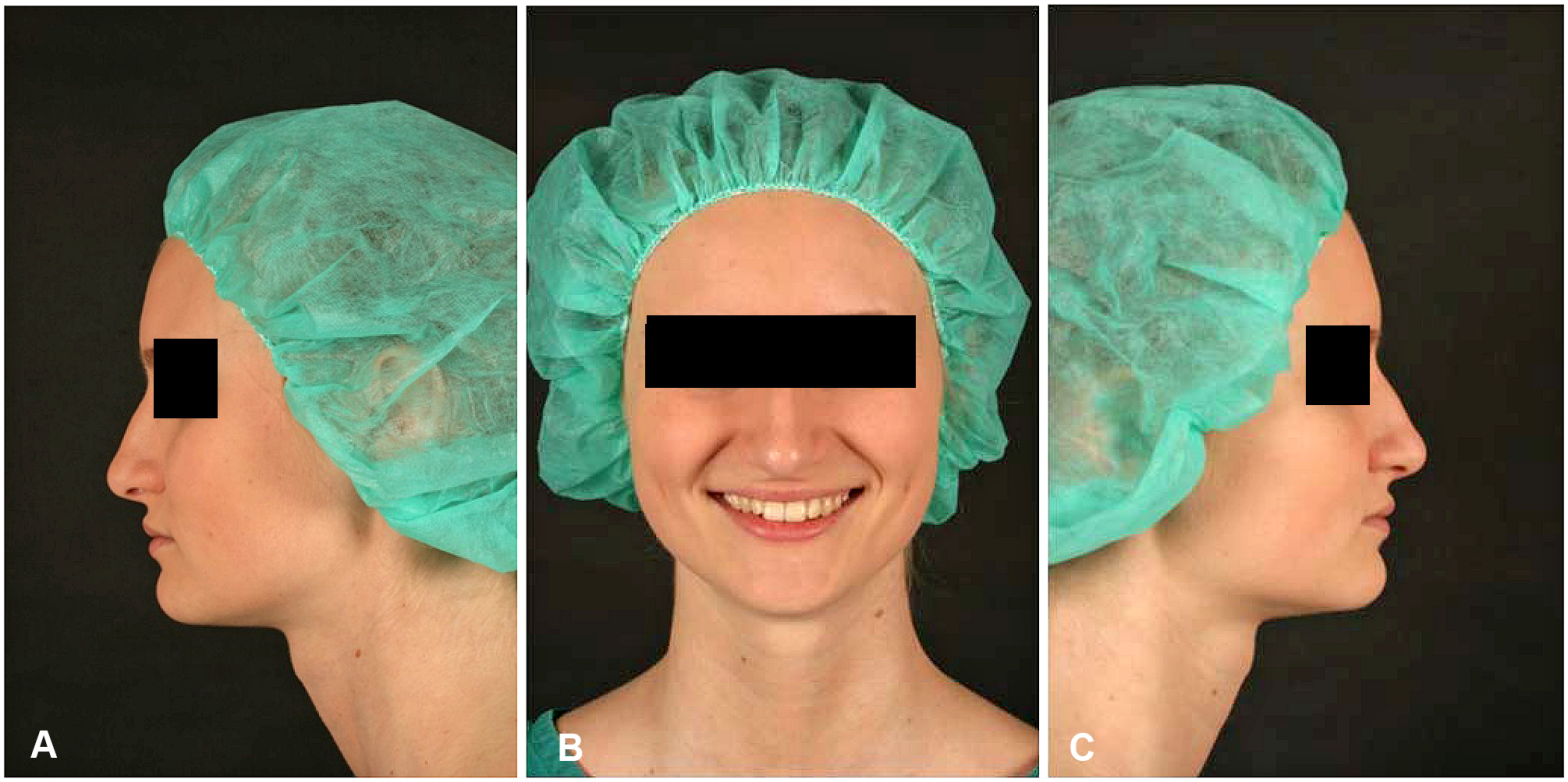
Three screenshots [left lateral (A), frontal (B), right lateral (C)] of one Class I control person, presented (without black bars) to the raters in the video.

The videos were taken by slowly rotating the probands seated on a swivel chair from the left lateral view to the frontal, and then on to the right lateral view (S1 Video, Class I patient as presented to the raters). We recorded the face and neck. Rotation was to and from the frontal view, at which point the subject briefly smiled, took 7–8 seconds. The end of the presentation was a right lateral view for about one second (Fig 1).

To enable the raters to include facial movement in their evaluation, each subject recited a standard sentence during the rotation “One usually says cheeeeeese on a photograph, although I’m not fond of cheese at all.”

Video clips were presented to the raters without sound, to allow them to focus on their visual impression.

### Description of rater

We advertised and realized the experimental part of the study at the psychology faculty of the University Vienna. Most raters had an academic background. The raters’ age distribution was similar to that of the persons presented in the videos. The raters were unaware of the study aims, and thus fulfilled the requirements of naïve observers. The raters were asked to judge the presented faces according to twelve items (Table 1).

**Table 1:** Seven point likert scale, and 12 items in sequence of presentation to the raters.

1	2	3	4	5	6	7
Absolutely	Very much	Quite	Neither/nor	Quite	Very much	Absolutely
ugly				beautiful		
unsuccessfull				successful		
unpleasant				pleasant		
unfaithful				faithful		
unattractive				attractive		
unintelligent				intelligent		
aggressive				good-natured		
unhealthy				healthy		
Inhibited				confident		
unerotic				erotic		
brutal				gentle		
dominant				flexible		

#### Rating procedure

The video clips of the study subjects were presented to the raters only once via a MS Powerpoint presentation on a computer screen. As per Rhodes *et al*. [13] the raters were instructed to assess the video clips spontaneously and intuitively with respect to the aesthetic dimensions and personality traits, which were evaluated by subjective intuitive perception using a seven-point Likert scale (Table 1). The presented material was viewed in small groups of about 10 to 15 raters without interaction between them.

In every session the first subject presented was always a control group (Class I), to establish a benchmark, provide an icebreaker, and provide the raters with a potential “anchor”.

This “warming up” was followed by the alternated presentation of 7–8 seconds video clip of the various classes of dysgnathia, varying also pre and post surgery videos to avoid sequence effects.

The video started automatically after a screen with the text “The video will start in 5 seconds …”. After the presentation, each video clip faded out and the following instruction appeared: “Now please rate the person on the video clip using the twelve pairs of traits.”

The intuitive rating process was carried out without the face on the screen. Raters paced the time they needed for their assessment. On average, the viewing and assessment of each video took about 30 seconds. To minimize systematic distortions during the assessment step, raters received a folder with a predetermined sequence of assessment sheets.

The first impression was considered important, therefore the raters were not permitted to modify previous ratings.

### Data processing and statistical analyses

Based on 16 (probands) x 2 (pre/post) x 156 (raters) x 12 (items) we should have obtained 59.904 ratings. We had one dropout among the raters. The complete data from the one proband always shown as first in a presentation series (as anchor and icebreaker) was removed from the data set, leaving over 55.000 entries for analysis. We used the statistical software IBM SPSS 22, an alpha of p < .01 was considered significant.

For depicting sociodemographic characteristics of the raters descriptive-statistical methods were used (frequencies, mean, standard deviation). Chi-square tests were employed for comparisons for categorical data and t-tests for independent samples for continuous data. General linear models (GLM) for repeated measures were applied to analyze data in order to establish whether groups of dysgnathia (class II, class III, Laterognathia as well as the norm group class I) differed in the traits rated over time (videos pre- and post-surgery). Additionally interactions between group and time were used to interpret different time courses of the four groups. Post-hoc-tests (Bonferroni) were calculated for items revealing significant group main effects. To evaluate the effect of differences identified by means of the GLM, partial eta^2^ was calculated, with values ≥.01 indicating a small, ≥.06 a medium, and ≥.14 a large effect.

## Results

### Probands

**Fig 2:**
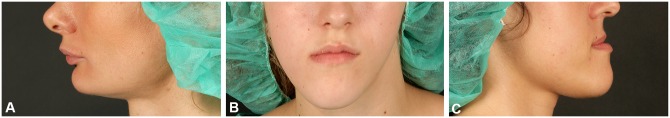
Screenshots of three dysgnathic patients [Class II (A); Laterognathia (B), and Class III (C)], presented to the raters in the video.

We presented videos from before and after orthognathic treatment from 4 female controls (Class I, Fig 1, and S1 Video) and 12 female dysgnathic patients (Class II, III, Laterognathia, (Fig 2).

The patients’ age at treatment start (before fixing of the orthodontic appliances) ranged from 15.5 yrs to 33.2 yrs (23.1 yrs ± 5,8, Mean ± SD). The age of the Class I controls in the first video ranged from 21.9 yrs to 27.7 yrs (24.1 ± 2.3, mean ± SD). The second video after the end of the treatment was taken after 3.2 ± 0.75 (mean ± SD) yrs in the patients, and 3.9 ± 0.19 (mean ± SD) yrs in the Class-I controls.

### Raters

We recruited 156 raters, one dropped out. Of the remaining 155 raters 83 were female (53.5%) and 72 were male (46.5%). The age ranged from 19 yrs to 42 yrs (24.83 yrs ± 3.0, mean ± SD). The raters body weight was from 39-108kg (67.8 kg ± 12.3), the height range was from 150–193 cm (173.6 cm ± 8.9; mean ± SD).

The marital status was single (N = 59; 38.1%), with spouse (N = 94; 60.6%), and 2 (1.3%) without information. The distribution of the highest educational level was “below university access” N = 1, (0,6%); “university access” N = 73; (47.1%); and “academic degree” N = 81 (52.3%). Among the academic raters 50 were psychologists, 102 indicated a different field, 3 provided no statement. 112 (72.3%) were Austrians, 35 (22.6%) were of German nationality and 8 (5.1%) came from other countries.

Only occasionally we observed significant differences in the pre/post analyses in single items between female and male raters, however the effect size was very low in all cases (Eta^2^ max = 0.048, data not shown).

### Ratings

Table 2 summarizes the ratings before and after treatment, and provides the statistical analysis of the impact of the surgical intervention in general, the difference between the individual Classes, and different surgery related changes between the four Classes.

**Table 2:** Statistics by generalized linear models.

Item No.	Items, in the presented sequence	See also Figure	Time point	Class I^a^ *M (SD)*	Class II^a^ *M (SD)*	Class III ^a^ *M (SD)*	Lat.gnath.^a^ *M (SD)*	Source of variance ^b^					
								*P*_*pre/pos*_	*Eta*^*2*^	*P*_*classes*_	*Eta*^*2*^	*P*_*IA*_	*Eta*^*2*^
1	ugly /beautiful	Fig 3A	pre	4.18 (0.94)	3.24 (0.93)	4.01 (0.98)	3.52 (0.98)	< .001	.218	< .001	.160	< .001	.143
			post	4.12 (1.02)	3.63 (0.92)	4.81 (0.80)	3.97 (0.87)						
2	un- /successful	Fig 3B	pre	4.71 (0.85)	3.78 (0.90)	4.38 (0.86)	3.98 (0.85)	< .001	.157	< .001	.149	< .001	.048
			post	4.76 (0.90)	4.14 (0.85)	4.88 (0.83)	4.34 (0.74)						
3	un-/pleasant	Fig 3C	pre	4.91 (1.00)	4.11 (0.82)	4.49 (0.99)	4.64 (0.89)	< .001	.100	< .001	.092	< .001	.147
			post	4.95 (1.06)	4.48 (0.93)	5.32 (0.84)	4.54 (0.81)						
4	un-/faithful	Fig 4A	pre	4.81 (0.94)	4.78 (0.87)	4.61 (0.86)	4.89 (0.87)	.001	.019	.156	.009	.056	.013
			post	4.87 (0.88)	4.96 (0.89)	4.77 (0.89)	4.87 (0.76)						
5	un/attractive	Fig 3D	pre	4.14 (0.96)	3.15 (0.86)	4.00 (0.99)	3.52 (0.96)	< .001	.192	< .001	.189	< .001	.149
			post	4.06 (1.05)	3.48 (0.91)	4.81 (0.83)	3.94 (0.89)						
6	un /intelligent	Fig 3E	pre	4.95 (0.90)	4.17 (0.95)	4.63 (0.90)	4.52 (0.86)	< .001	.120	< .001	.080	< .001	.037
			post	4.99 (0.89)	4.52 (0.95)	5.03 (0.82)	4.78 (0.79)						
7	aggressive /good-natured	Fig 4B	pre	4.88 (1.03)	4.82 (0.82)	4.56 (0.81)	5.08 (0.85)	< .001	.055	.361	.005	< .001	.093
			post	4.96 (0.92)	5.09 (0.87)	5.16 (0.83)	4.94 (0.72)						
8	un-/healthy	Fig 3F	pre	5.23 (1.03)	4.43 (1.04)	5.08 (0.99)	4.43 (1.07)	< .001	.062	< .001	.123	< .001	.048
			post	5.17 (1.04)	4.58 (1.02)	5.48 (0.91)	4.76 (0.96)						
9	inhibited/confident	Fig 5A	pre	4.89 (1.01)	3.50 (0.77)	4.44 (0.91)	3.49 (0.95)	< .001	.119	< .001	.385	< .001	.038
			post	5.02 (0.97)	3.68 (0.82)	5.01 (0.83)	3.96 (0.82)						
10	un-/erotic	Fig 3G	pre	3.38 (0.92)	2.56 (0.87)	3.33 (1.02)	2.89 (0.90)	< .001	.098	< .001	.139	< .001	.078
			post	3.30 (1.05)	2.83 (0.96)	3.91 (0.94)	3.19 (0.91)						
11	brutal/gentle	Fig 4C	pre	4.76 (0.87)	4.71 (0.77)	4.49 (0.70)	5.01 (0.79)	.001	.017	.013	.018	< .001	.087
			post	4.67 (0.83)	4.98 (0.83)	4.89 (0.79)	4.83 (0.77)						
12	dominant /flexible	Fig 5B	pre	4.19 (0.97)	4.62 (0.80)	4.06 (0.77)	4.91 (0.79)	.084	.005	< .001	.158	< .001	.042
			post	4.11 (0.94)	4.78 (0.81)	4.09 (0.76)	4.56 (0.78)						

^a^ Mean M, standard deviation *SD* for items rated

^b^ *P*_*pre/post*_ probability “pre/post treatment differences”; *P*_*classes*_ difference between classes; *P*_*IA*_ probability interaction pre/post x group; *Eta*^*2*^ *Effect size*

#### Aesthetic traits and those with similar patterns

**Fig 3:**
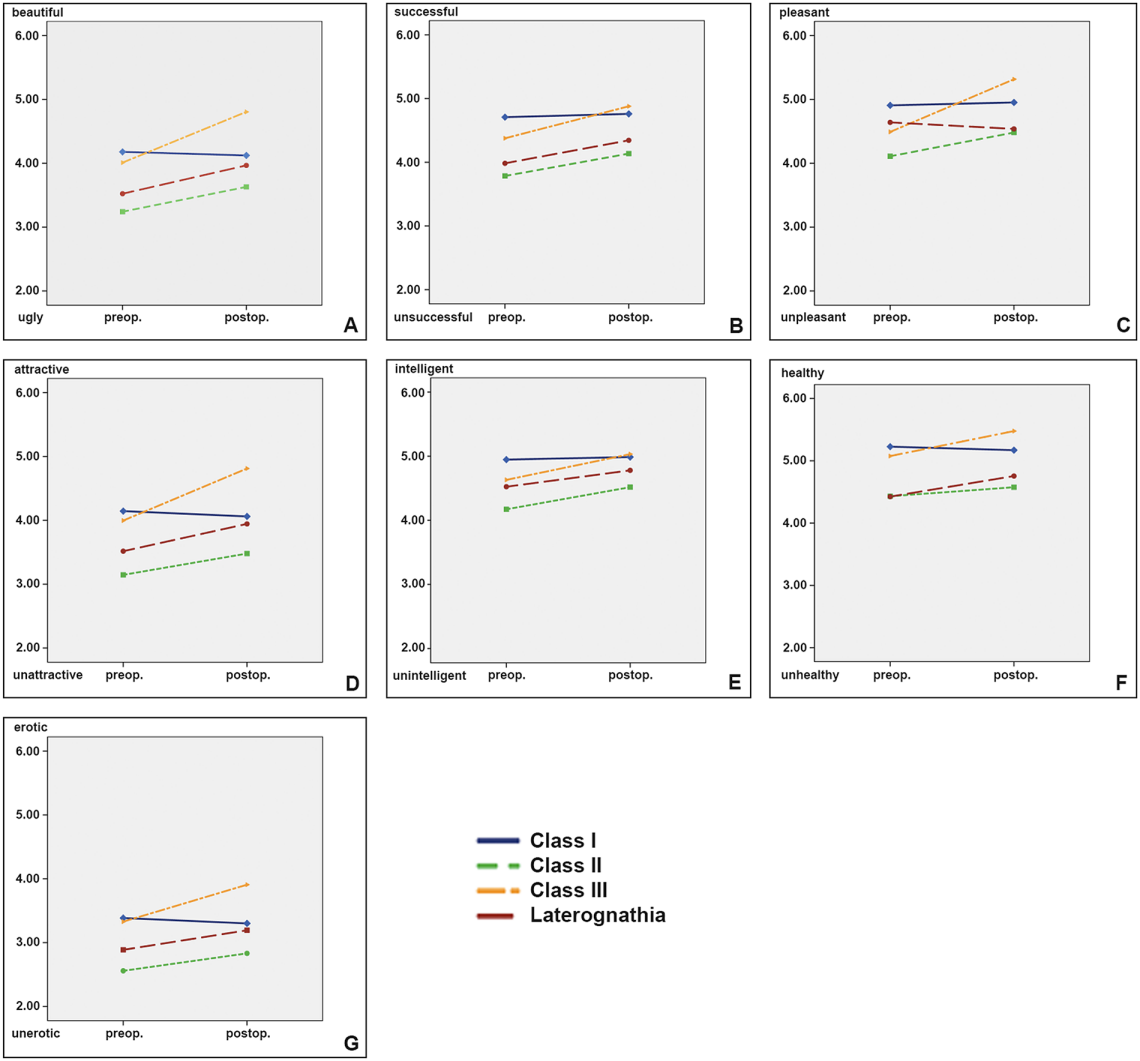
Aesthetic traits and those with similar pattern [ugly /beautiful (A), un-/successful (B), un-/pleasant (C), un- /attractive (D), un-/intelligent (E), un- /healthy (F), un- /erotic (G)].

Fig 3 shows the results of the aesthetic items and those which revealed a very similar pattern before and after surgery.

Before surgery the control patients (Class I, without surgical and orthodontic intervention) had the highest ratings for all traits, and remained at the same level at the second assessment. All three dysgnathic classes showed an intervention related improvement of the ratings for the respective trait. Class III patients revealed the largest benefit from surgery, in some traits the scores were above the controls (Fig 3) Class II patients had the smallest benefit.

#### Personality traits without differences between the classes

**Fig 4:**
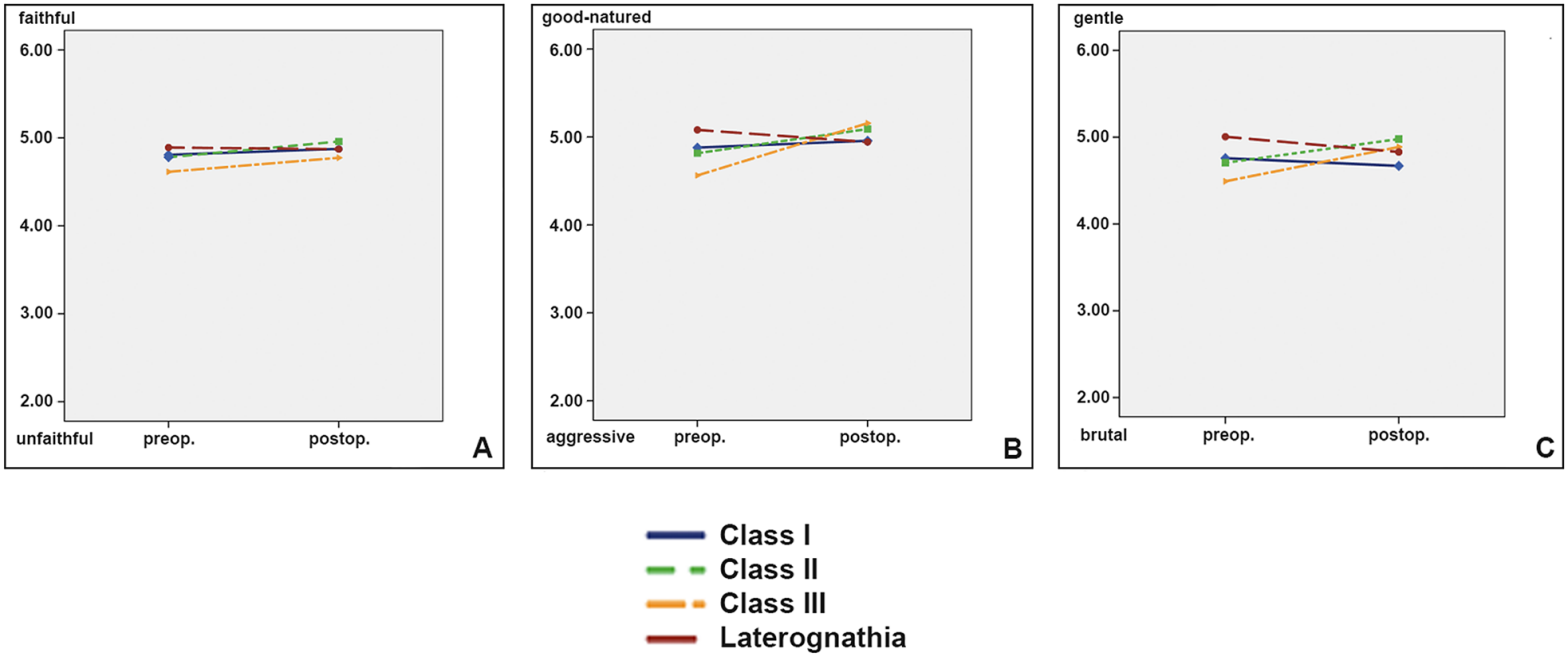
Personality traits with little differences between the classes, and low impact of the treatment [un- /faithful (A), aggressive /good-natured (B), brutal /gentle (C)].

Fig 4 shows the results of personality items with only little difference between the classes.

Many raters provided the feedback that they find it very difficult to rate faithfulness on a short video. The differences between the control patients (Class I, without surgical and orthodontic intervention) to the dysgnathic patients showed only very mall effect sizes (see Table 2 item 4,7,11) whether before nor after the surgical procedure (Eta^2^ max < 0.09).

#### Personality traits with differences between the classes

**Fig 5:**
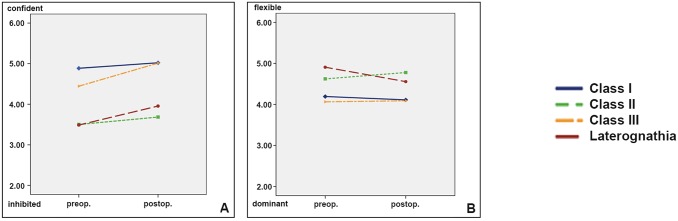
Personality traits with considerable differences between the classes before and after surgical intervention [inhibited /confident (A), dominant /flexible (B)].

Fig 5 shows the results of two personality traits with considerable differences between the classes.

For the rating of confident /inhibited, before surgery, all dysgnathic patients received lower rates compared to the controls (were perceived as less confident). The ratings after surgical intervention revealed a considerable impact towards more confidence in all three dysgnathic classes, Class II patient post op ratings revealed the lowest benefit (Fig 5A).

Concerning dominant/flexible the Class III patients were similar to Class I and revealed almost no surgery related changes. The surgery related difference for patients with Laterognathia and Class II were small (Fig 5B).

## Discussion

Orthognathic surgery improves the assignment of aesthetic and some specific personality traits by naïve raters.

To focus the raters’ judgement on the physical characteristics related to and changed by the surgical intervention, we minimized distracting physical signs as much as possible, e.g. we recruited adult patient aged from 18 to 33 years, without noteworthy signs of aging. To minimize distraction of the raters by impressions other than the physical face we produced the videos from the patients in uniform conditions, i.e. dressed in a green outfit as used I the operation room, with the hairs completely covered by a green surgical cap. The patients were seated on a swivel chair, to minimize any bias related to individual elegance or sportiness. Also for standardizing the clips, all patients spoke the same sentence to record their lip movement. The content and length of the video was the same in all cases.

It is known that women as raters tend to rate male faces more ambiguously. For example Hönn *et al*. [11] described that women’s perception of the attractiveness of male faces is markedly influenced by the women’s menstrual cycle. For this, and because 2/3 of the orthognathic patients are female and the rating of aesthetics is more consistent with females faces [2], we picked female patients only. Interestingly we observed no or negliable differences between our male and female raters.

Also for other reasons the selection of the patients and therefore the videos was not at random. We preferentially picked typical members of “their class”. The arbitrary selection of patients renders a comparison of the ratings “before surgery” with results from other studies meaningless. The overall study goal was to measure the change of perception between “before” and “after” surgery per trait and class, and then compare these changes to typical age related physical signs in orthognathic faces (controls, Class I).

Most of the raters had an academic background and were grown up in German speaking countries, therefore the overall study results may be most representative for this group and should not be generalized to the whole population. We matched the age of raters to the patient age by recruiting preferentially students in the same age range, because the perception by peers with similar age is most relevant if aesthetic traits are rated. Possibly—if the research question would specifically relate to work place and career, the judgement of personal traits by older raters, (reflecting the boss-employee situation) might be slightly more representative than our actual setting.

During the presentation the pre and post videos from a specific person were presented in alternated order, to minimize a possible systematic bias induced by the rating of a familiar (recognized) face.

To set an anchor, one Class I face was always shown first, to set a Class I face as benchmark for the raters. The starting (anchoring) ratings—largely influenced by the daily mood and conditions the raters brought into the experiment—were removed from the data set before data analysis.

Methodologically our study differs from previous investigations where still photographs were rated. However as discussed later the obtained video based ratings are similar to those from still images, for the various aesthetic and investigated personal traits [4].

After the drop out of one rater, and following the removal of the ratings from the one anchoring face, the remaining ratings of “before” and “after” the surgical intervention allowed for valid conclusions.

Because statistical significance is highly dependent on sample size, we also calculated the effect size of the differences to estimate their relevance. The use of 155 raters in the present study and analysis for significant differences may cause an overestimation of significance due to sample size. Effect sizes (small, moderate, large) are independent of sample size and thus provide a useful additional measure for the clinical relevance of the findings. Therefore in Table 2 we provide both significance (p) and effect size (Eta^2^).

### Rating-results

We prepared the rating situation to obtain the raters perceptions on basis of meagre information collected in 7–8 seconds. Our Results “Pre intervention” show both, traits with remarkable differences between the dysgnathic classes or deviations from Class I, or alternatively we identified traits where the classes were rated very similarly. In the latter many if not all raters felt unable to judge this specific trait on basis of a short single presentation and therefore reached a mean close to each other (Fig 5).

If the traits showed a difference between the classes, this may be due to our arbitrary selection of the patients’ faces. There is an academic dispute whether the perception of Class II or alternatively Class III is perceived more or less beautiful. With four arbitrarily picked faces per class we cannot, and we did not intend to participate in this discussion. The focus of our study was on the differences attributable to the surgical intervention. Typically orthognathic surgery aims to approximate the anatomy of Class I faces, simply because in our society Class I faces represent the social benchmark to which dysgnathic patients aspire.

Our data reveal that the expectations have been met. After the orthognathic surgery intervention the post OP ratings shifted towards Class I. Among Class I faces the mean ratings pre and post revealed almost no particular differences. This underlines the trustworthiness of our measuring system, and it corroborates the relevance of the various differences in the respective dysgnathic patient groups (classes). Our mean ratings of Class II, III and Laterognathia patients, may fairly reflect the surgery related changes in the face perception.

#### Aesthetic and associated traits

Fig 3 shows the results of 7 items with a very similar pattern before and after the surgical intervention. These items were: ugly /beautiful, un-/successful, un-/pleasant, un-/attractive, un-/intelligent, un- /healthy, un- /erotic.

Class I did not reveal significant changes over the observation time, matched with the average treatment time for dysgnathic patients.

Class II patients benefitted very little from the orthognathic intervention. Possibly because only two out of four patients received bimaxillary osteotomy the changes from before to after the intervention were not dramatically. Furthermore in our videos the lateral views which enhance the perception of dysgnathia were only a small part of the presentation.

In the last centuries females with a slightly retruded chin were the beauty ideal, rather than those with a prominent chin. Possibly with the increasing impact of gender issues and female emancipation, nowadays a straight face with a prominent chin is increasingly perceived attractive. This may explain why in our patient sample Class III patients—after orthognathic surgery—performed better than the benchmark (Class I). Our Class III patients had the steepest improvement, they benefited most from the intervention, postoperatively they were rated better than controls (Class I).

It is possible that Class III patients benefited also from the presentation in a videoclip instead of still photographs. While the rating on photographs focusses on the rating on the lateral view, we presented the faces of our patients moving from right lateral to frontal to left lateral. Therefore the time of lateral view perception was relatively short, in addition when presenting the frontal view our probands smiled at the rater, which may have contributed to higher ratings for aesthetic rates for beauty and attractiveness [4].

Laterognathic patients benefitted considerable from the orthognathic intervention All these patients received bimaxillary surgery, which possibly corroborates the concept that more surgery implies also more impact on the viewers’ postoperative perception. In Laterognathic patients the symmetry hypothesis on beauty and attractiveness proposed by Little *et al*. [18] may have had the greatest impact on video presentations because a frontal view pronounces the typical facial asymmetry of Laterognathic patients.

#### Personality items

Fig 4 depicts the personality traits, which have in common that they did not differ largely from each other, neither before nor after the surgical intervention, corroborating earlier findings on still photographs [4]. Because many raters actively complained, that it is difficult to rate faithfulness on a short video, it is no surprise that the mean of the ratings lies in the middle of the scale, without substantial surgery related changes (Fig 4, Table 2, Eta^2^ < 0,1).

Fig 5 shows the mean ratings for the traits which differed considerably between the classes (Table 2, Eta^2^ 0.385) indicating that the physical face appearance of a dysgnathic class conveys a stereotypical perception of these two traits. Concerning the trait confidence, Class I and Class II showed minimal changes. Class III and Laterognathic patients revealed a substantial difference between pre and post rating, e.g. indicating an therapy related increase of confidence (Table 2, Eta^2^ = 0.119).

In contrast, the rating of dominant/flexible did not change after the surgery. This may be surprising, as the expectation is that retrognathic (Class II) patients are perceived less flexible and more dominant after transposition of the lower jaw forwards, and Class III patients are perceived less dominant after surgically retro-transfer of the lower jaw. Our results on video clips do not disagree with these expectations but also do not corroborate them. Most likely the presentations in a video clip with views from lateral and frontal, and with the patients smiling, seem convey more information to the rating naïve viewer, so that the rating is less influenced by stereotypes.

### Faces in motion or still images

As different regions of the brain are known to be activated for the evaluation of visual stimuli, the biology of perception depends on whether an object is in motion or not. Kessler *et al*. [19] described relevant brain regions to process dynamic stimuli. The frontal and parietal cortex, more specifically the bilateral superior temporal sulcus, the visual area V5, the fusiform gyrus, the thalamus, and other become activated. In contrast static stimuli are processed in the medial prefrontal cortex. Consistent to this Johnston *et al*. [20] showed that still images are mainly processed in the frontal regions of the human brain. We propose that video clips (faces in movement) compared to still images are perceived differently, the additional mimics presented in videos ameliorate the perception of classical stereotypes. E.g. the additional information conveyed by moving images, reduces the raters’ perception of self-confidence (Table 2).

To our knowledge we are the first to investigate the perception of dysgnathic faces based on videos instead of still images. As surgical impact measurements of confidence and other traits in our Class III faces were more marked in our previous study [4], rating of static faces may enhance the perception of common stereotypes.

## Conclusion

Motion might mitigate the perception of common stereotypes, but cannot entirely offset the negative effect of asymmetry on face perception. Orthognathic surgery can improve the perception of all dysgnathic patients. Our study indicates that Class III patients benefit most.

Specific personality traits (e.g. faithful) apparently are not stereotypically associated with a specific face. Out of the 12 tested items seven were perceived similarly between the classes before treatment start, and the orthognathic surgery had a similar impact on these items, which may indicate a common stereotypic root in the perception of these items (beautiful, successful, pleasant, attractive, intelligent, healthy, and erotic).

## Supporting information

S1 Video Class I patient as presented to the raters. (MOD)
